# 

**Published:** 1887-08

**Authors:** 


### The Chicago Medical Journal and Examiner.

### PUBLISHERS' ANNOUNCEMENT.

The Chicago Medical Journal and Examiner is the oldest of the Western medical journals, and it will be the aim of the publishers to make it the best. The volumes begin with January and July. The subscription price is $3.00 per annum, or $1.50 per volume; single copies, 25 cents. Subscriptions received at any time. Address the publishers,

The Clark & Longley Company,
308-316 Dearborn street, Chicago, Ill.

Volume LV.] AUGUST, 1887. [Number 2.

### The CHICAGO MEDICAL JOURNAL AND EXAMINER

EDITOR:
S. J. JONES, M.D., LL.D.

COLLABORATORS:

CHICAGO:

PROFESSOR E. ANDREWS, M.D., LL.D. PROFESSOR C. FENGER, M.D.
PROFESSOR J. BARTLETT, M.D. PROFESSOR J. N. HYDE, A.M., M.D.
PROFESSOR W. T. BELFIELD, M.D. PROFESSOR E. F. INGALS, A.M., M.D.
PROFESSOR F. BILLINGS, M.D. PROFESSOR W. W. JAGGARD, A.M., M.D.
PROFESSOR W. H. BYFORD, A.M., M.D. PROFESSOR F. S. JOHNSON, A.M., M.D.
PROFESSOR L. CURTIS, A.M., M.D. PROFESSOR H. A. JOHNSON, M.D., LL.D.
PROFESSOR N. S. DAVIS, JR., A.M., M.D. PROFESSOR C. W. PURDY, M.D.
PROFESSOR O. C. DEWOLF, A.M., M.D. PROFESSOR C. G. SMITH, A.M., M.D.
PROFESSOR E. C. DUDLEY, A.B., M.D. PROFESSOR E. WING, A.M., M.D.

MILWAUKEE, WIS.
PROFESSOR N. SENN, M.D.

WASHINGTON, D. C.:

S. O. RICHEY, M.D.

UNITED STATES ARMY:
SURGEON D. L. HUNTINGTON, M.D.

MARINE HOSPITAL SERVICE:
SURGEON S. T. ARMSTRONG, M.D.

UNITED STATES NAVY:
MEDICAL DIRECTOR J. M. BROWNE, M.D.
MEDICAL DIRECTOR A. L. GIHON, A.M., M.D.

